# Effects of a Cognitive Rehabilitation Programme on the Independence Performing Activities of Daily Living of Persons with Dementia—A Pilot Randomized Controlled Trial

**DOI:** 10.3390/brainsci11030319

**Published:** 2021-03-03

**Authors:** María Jiménez Palomares, María Victoria González López-Arza, Elisa María Garrido Ardila, Trinidad Rodríguez Domínguez, Juan Rodríguez Mansilla

**Affiliations:** 1 ADOLOR Research Group, Department of Medical-Surgical Therapy, Medicine Faculty, Extremadura University, 06006 Badajoz, Spain; mariajp@unex.es (M.J.P.); mvglez@unex.es (M.V.G.L.-A.); jrodman@unex.es (J.R.M.); 2 ROBOLAB Research Group, Medical-Surgical Therapy Department, Nursing and Occupational Therapy Faculty, Extremadura University, 10003 Cáceres, Spain; trdomin@unex.es

**Keywords:** Occupational Therapy, cognitive stimulation, functionality, independence

## Abstract

Background: In all types of dementia, cognitive abilities are affected, behaviour is altered and functional capacity is progressively lost. This cognitive deterioration manifests in the decrease of abilities required to perform the activities of daily living (ADL). The objective of this pilot study was to assess the effect of an Occupational Therapy programme based on the training of ADL through cognitive stimulation on the independence of ADL of persons with dementia. Methods: Institutionalized older adults with major neurocognitive disorder or dementia (N = 58) were randomly allocated to receive either the Occupational Therapy ADL cognitive stimulation programme or conventional Occupational Therapy. The cognitive level and the independence level performing ADL were measured at baseline (week 0), after 5 weeks of treatment (week 5) and after 6 weeks of follow up (week 12). A value of *p* < 0.05 and α = 0.0025 (Bonferroni correction) was considered as statistically significant. Results: The results obtained showed improvements in the level of independence performing ADLs in the intervention group compared to the control group (*p* = 0.006). The improvements were seen in relation to feeding (*p* = 0.001), dressing (*p* = 0.005) and bladder and bowel incontinence (*p* = 0.003), the changes observed in feeding are statistically significant. However, those improvements were not maintained after the follow up period. There were no significant changes in relation to the cognitive level (*p* = 0.741). Conclusions: Occupational Therapy based on ADL cognitive stimulation can have a positive effect, increasing the independence of subjects with major neurocognitive disorder or dementia who are institutionalised.

## 1. Introduction

The Alzheimer’s Disease International report, published in 2016, estimated that approximately 46 million people in the world have dementia [1,2], also known as major neurocognitive disorder [3,4]. If the ageing trend continues as at present, 131 million people with dementia would live in 2050 and two thirds of those people will be from developing countries [1,2]. Moreover, dementia is one of the main causes of institutionalisation. In particular, the rate of institutionalisation in Spain is 10.5%; and 36% of people with disability that are in residential homes have dementia [1,2].

In this disorder, regardless of its aetiology, the cognitive abilities are affected, behaviour is altered and functional capacity is progressively lost [3,4,5]. The research available has indicated that each type of dementia can present specific symptoms that can categorise the disease with more or less accuracy. Considering the origin of the symptoms, they can be classified into three groups—intellectual-cognitive, physical and functional [4,6]. Among the last ones, difficulties or limitations in instrumental activities and basic activities of daily living (BADL), incontinence, mobility problems and swallowing alterations are included.

The cognitive deterioration implied in the major neurocognitive disorder is normally shown by a decrease of the abilities required to appropriately carry out activities of daily living (ADL) [7]. Advanced activities are the first affected, followed by instrumental activities. Advanced activities are those complex activities that allow us to perform our roles, leisure activities and which lead to self-fulfilment. If the cognitive deterioration continues, even the basic activities of daily living can be affected [8]. The number and the type of activities affected as well as the limitation of their performance, varies according to the severity of the cognitive deficit, the personal situation and the environment of the person [7]. The important aspect of this function loss is the fact that it leads to disability, carer overload and the increase of institutionalisation rates and economical expenditure [2].

In recent years, non-pharmacological treatments have been used in combination to drug therapy in order to maintain independence [9,10]. These therapies have multiple objectives such as to stimulate, maintain or improve mental abilities and cognitive performance, to improve or maintain functionality and independence to perform activities of daily living and to ensure and increase personal autonomy [8,11]. Patient-centred non-pharmacological therapies include cognitive stimulation, cognitive training, cognitive rehabilitation and ADL training among others [8,12].

Scientific evidence suggests that rehabilitation of the cognitive function is possible from the biological perspective. Cognitive stimulation approaches can have therapeutic benefits for patients with mild to moderate dementia delaying the cognitive deterioration [8,13,14,15]. Therefore, the evidence supports that the use of this type of approach improves the condition of the persons with dementia.

The treatment approaches used by Occupational Therapists to improve the limitations of the ADL performance can be classified in four categories [16]:

*Compensation approach*: this approach has three strategies: to change the way to carry out the activity, to adapt the objects involved in the task and/or to modify the environment.

*Reestablishment approach*: The intervention is focused on the level of deficit and it has the objective to restore the necessary abilities for the functional activities.

*Educational approach*: It is based on the education of the patient as well as the carers.

*Holistic approach*: It combines the three previously explained approaches.

The most recent research has shown that, in the field of dementia or major neurocognitive disorders, the approaches that have mainly been chosen are the compensation and educational approaches [13,17,18,19,20,21,22]. The compensation approach has focused mainly on modifying the environment or simplifying tasks [13,17,18,19]. The educational approach has trained the carers in the management of the problems faced during ADL performance [13,20,21,22].

The authors of this study have developed a programme, which is a treatment approach for the training of ADL through cognitive intervention. The programme is based on the re-establishment of the cognitive functions implied in the performance of basic activities of daily living.

The objective of the present study was to assess the effects of the Occupational Therapy ADL cognitive stimulation programme on the re-establishment of the cognitive abilities and the independence of persons with dementia or major neurocognitive disorders who are institutionalised.

## 2. Materials and Methods

### 2.1. Study Design

This pilot study was a double-blind randomized clinical controlled trial. The patients and the assessor were blinded. The patients and their carers did not know to which group they were allocated. They knew that they were receiving Occupational Therapy treatment but were not aware of what particular approach. In addition, they were not aware of the type of intervention they were receiving owing to their pathology. The assessor, an Occupational Therapist independent to the study, was not aware of the objective of the therapy, the treatments applied or to whom they were being applied to. The study was approved by the Bioethical Commission of the University of Extremadura in Spain (Registration number: 46/2012) and it was prospectively registered (Clinical Trials.gov, Identifier NCT02199353).

The sample consisted of 58 participants who were randomly allocated to either an intervention group or a control group. A computer random number generator was used to produce even allocation ratios within block sizes of three by group allocation through the Quick Cales application from GraphPad Software (San Diego, CA, USA). This randomisation list was held by an independent researcher (Occupational Therapist) who was unrelated to any aspect of the trial.

### 2.2. Participants

The target population were older adults diagnosed with dementia who were recruited during a three-month period. The sample consisted of 58 subjects institutionalised in ‘CARE’ residential homes (Extremadura, Spain). The written informed consent was signed by the legal guardians of the patients prior to the beginning of the study.

The inclusion criteria were: patients diagnosed with dementia by a physician specialising in geriatrics for at least one year according to the Diagnostic and Statistical Manual of Mental Disorders (DMS IV) criteria [3]; adults over the age of 60 who were institutionalised in the residential home at least 6 months before the commencement of the study; Barthel Index scores [23] greater than or equal to 40 points; Lobo’s Cognitive Mini Test scores (Spanish version of the Minimental Status Examination) [24] greater than or equal to 15 points; patients that were receiving Occupational Therapy in the residential home and were following the BADL intervention programme for at least 6 months; informed consent signed by the legal guardian. The exclusion criteria were patients who presented psychological or behaviour symptoms which were diagnosed by a doctor; Barthel Index scores less than 40 points; patients who were not receiving Occupational Therapy in the residential home or did not follow the BADL intervention programme for at least 6 months.

### 2.3. Interventions

**Figure 1:**
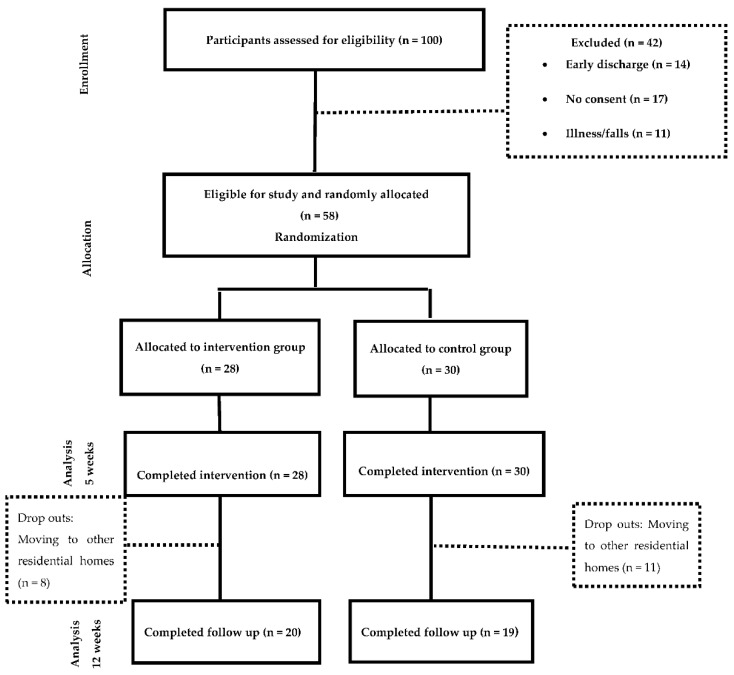
Flowchart of resident’s participation.

The sample was allocated to two groups (Figure 1)—the Intervention group, which received Occupational Therapy programme based on the training of ADL through cognitive stimulation and the control group, which received was based on a conventional Occupational Therapy intervention for the management of ADL deficits. Details of the intervention following the Template for Intervention Description and Replication (TIDieR) guidelines are provided in Supplemental Table S1. In addition, a comparison of both interventions can be found in supplemental Table S2 All participants continued with their routine medical complying with the beneficence and non-maleficence principles of bioethics. The study was conducted for 12 weeks;—five weeks of treatment, five weeks of follow up and two weeks of measurements.

### 2.4. Data Collected and Outcome Measures

The following data were collected: Socio-demographic data, type of dementia, concomitant treatment and outcome measures. The primary outcome measure was the dependency level for the performance of BADL and the secondary outcome measure was the cognitive level. The cognitive deficits were assessed with the following scales:

The Global Deterioration Scale (GDS) [25]: This scale assesses the level of cognitive deficits. The results can be rated from 0 to 35 and indicate: GDS 1—no cognitive deficit (30–35), GDS 2—very mild cognitive deficit (25–30), GDS 3—mild cognitive deficit (20–27), moderate cognitive deficit (16–23), moderate to severe cognitive deficit (10–19), severe cognitive deficit (0–12) and very severe cognitive deficit (0).

The Lobo’s Cognitive Mini Test (LCMT) or the Spanish version of the Minimental Status Examination [24]: This screening test quantifies the cognitive level of a person. The total score ranges from 0 to 35 and the results indicate: No dementia (30–35), borderline (29–25), dementia (less than 24), mild dementia (24–20), moderate dementia (19–15), severe dementia (14 or less).

In order to assess the BADL, the Barthel Index was used. The original Barthel Index consists of 10 items, which are scored in five-point increments, with possible sum-scores ranging from 0 (totally dependent) to 100 (totally independent) [23]. This allows the assessment of any changes in each basic activity area evaluated with this scale.

### 2.5. Statistical Analysis

A descriptive analysis of the characteristics and the baseline measurements of the 58 participants was performed, showing the distribution of categorical variables and centralisation measures and the statistical dispersion and position of the continuous variables. The normality of the continuous variables was analysed with the Kolmogorov-Smirnov test. The differences regarding the baseline characteristics between participants of the intervention group and participants of the control group were studied through the Pearson χ^2^ test and Fisher’s exact test (when at least 20% of the boxes in the contingency table showed a frequency below 5). In addition, the t-Student test was used for normal continuous variables and the Mann-Whitney U test was used for non-parametric variables. The following outcome measures were also analysed: The cognitive level, the scores and level of dependence of Barthel Index, the difficulties performing ADL and the cognitive function of the control group versus the intervention group after the treatment and in the follow up. They were all analysed applying the t-Student, the Pearson χ^2^ and the ratio of verisimilitude tests. The SPSS Statistical software, 21 version for Windows was used for the analysis, considering statistically significant a value of *p* < 0.05. In addition, the Bonferroni correction was used since there were twenty separate comparisons and the significance value considered was α = 0.0025. The Little’s test was used to verify whether the dropouts of the sample were missing completely at random (MCAR). In order to analyse if the Barthel Index scores of the intervention group and the control group were related to the cognitive deficit or the type of treatment, an analysis of variance (ANOVA) was carried out when the variables were continuous and had normality. The Pearson’s χ^2^ and the Fisher’s test were done when the variables were categorical.

No formal power calculation was carried out since the study relied upon the availability of the patients institutionalised in the residential homes to participate. Based on statistical guidelines [26] and previous studies of our research group, it was anticipated that a minimum of 25 participants per group might be recruited and would be enough to justify the use of the statistical methods actually employed.

## 3. Results

A total of 58 subjects participated in the study, 28 were allocated to the intervention group (48.3%) and 30 were allocated to the control group (51.7%). The measurements were taken at week 0 and after the treatments were completed (week 5). The follow up measurement (week 12) was completed with 39 participants, where 20 of them were in the intervention group and 19 in the control group. The dropouts were due to subjects moving to other residential homes.

Socio-demographic data can be seen in Table 1. The results of the Chi Square test showed homogeneity of the main baseline characteristics of both groups (Table 2).

The results showed that only 69% of the subjects could feed themselves independently, 96.60% were dependent for bathing, at least 60.30% needed some assistance for dressing and only 8.60% were dependent for their personal hygiene. A percentage of 46.60 were urine continent and 39.76% were stool incontinent. Over half of the subjects needed assistance to go to the toilet, being 6.90% totally dependent. Regarding bed to chair transfers and mobility, 53.40% of the participants were independent, although more than half were dependent to go upstairs and downstairs.

**Figure 2:**
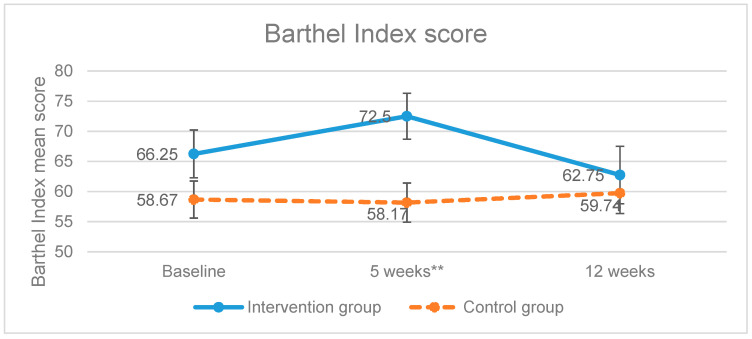
Evolution of the Barthel Index scores. ** *p*-value < 0.01; Error bars represent standard errors.

Once the treatment was completed, the intervention group increased the scores of the level of dependence of BADL variable, showing a significant improvement (*p* = 0.006) as higher scores mean higher degree of independence (Figure 2). On the other hand, the control group scored less points compared to the baseline measurements. The improvement of the intervention group was not maintained in the follow up measurement (week 12) as the scores obtained had a tendency to be equal.

In addition, in order to assess which specific activities improved, an analysis of each BADL assessed in the Barthel Index was conducted. The results showed statistically significant differences that are detailed in supplemental Table S3. After the treatment, the intervention group improved the performance of some BADL, which are reflected in an increase in the percentage of participants who were independent in each activity or a decrease in the percentage of dependency.

The participants of the intervention group needed less assistance for feeding (*p* = 0.001), they were less dependent on dressing (*p* = 0.005) and they had less incontinence (*p* = 0.003) of both urine and stools than the control group. The improvements found in relation to feeding were statistically significant.

Unlike the BADL, the statistical significance between the intervention group and the control group regarding the item ‘go upstairs and downstairs’ can be observed in the follow up measurement but not immediately after the treatment (*p* = 0.157). The results of the measurement taken at week 12 showed that more participants from the control group were independent or needed help to accomplish this task (*p* = 0.002). In the follow up period, the improvements achieved by the intervention group had a tendency to be equal or disappear.

As can be observed in Table 3, the ANOVA showed no statistically significant differences between the Barthel Index scores and the cognitive deficit or severity of the dementia (GDS).

Regarding the secondary outcome measure (cognitive level), no statistically significant differences in global cognitive status were observed between the two groups after the application of the treatments (*p* = 0.741). However, a maintenance of the cognitive level was observed.

The results of the Bonferroni correction, used for the analysis of the baseline and week 5 characteristics of the dropouts and the retained participants, showed no statistically significant differences between groups. Moreover, the results of the Little’s test suggested that the dropouts were missing completely at random.

## 4. Discussion

The objective of the present pilot study was to assess the effects of the Occupational Therapy ADL cognitive stimulation programme on the independence of institutionalised patients with dementia. Our results suggest that this treatment, which is based on a re-establishment approach and focuses on the rehabilitation of the cognitive function, improves functional capacity of persons with dementia. The intervention group showed a significant improvement in the performance of ADL. After completing the treatment, the participants of the intervention group maintained their cognitive level and increased their Barthel Index score improving their independence on feeding and dressing, their bowel and bladder incontinence and their ability to go upstairs and downstairs.

Scientific evidence has confirmed that dementia, or major neurocognitive disorder, limits the independence of the persons as well as their capacity to perform activities of daily living. The affected capacities will vary depending on the stage of the disease. In the mild to moderate stages, the main changes have an influence on ADL participation [8,27,28]. In our study we have observed that half of the participants of the intervention group presented a moderate dependency to perform ADL and only 1.70% was totally independent. Based on this data, we can consider that the intervention on functionality problems of persons diagnosed with dementia or major neurocognitive disorder needs to be a priority [8,10,13,29].

The relationship between cognitive impairment and the functions of daily living has been widely studied, especially when both are present in the diagnosis of the disorder itself. There are authors who establish a clear and evident correlation between cognition and functional capacity [30,31].

In contrast, in our study, when assessing whether the level of cognitive impairment influences the functional capacity of the participants, we found that there were no significant differences in the Barthel index scores in relation to the degree of cognitive deficit (GDS) (*p* = 0.262, *p* = 0.429). These results coincide with the theories that suggest that functional capacity is complex and indicate that cognitive measurements alone do not explain the deterioration of ADL functioning since there are other variables such as motor, sensory and perceptual alterations, social situations and comorbidities that also contribute to ADL performance [28,32]. It has also been evidenced that the ADL analysed in our study are the last to be lost in dementia [7,8]. In addition, these activities are cognitively simpler than instrumental ones [33] which can explain the results obtained.

A detailed assessment of the dependence degree is essential for the appropriate identification of the deficits and the prescription of the appropriate treatment [16]. Analysing the evidence in the literature, we can observe that the publications that study ADL use the Interview for Deterioration in Daily Living Activities in Dementia scale (IDDD) [17,18,19,34,35]. This scale is completed by the main caregiver according to the assistance required by their relative. Unlike these studies, BADL have been assessed in our research with the Barthel Index. This scale was used because the subjects were institutionalised and their caregivers could not complete the observational scales. This was due to the fact that their working shifts changed constantly which did not always allow them to provide their services to the same person. In addition, this scale has been validated and translated into Spanish and we had enough professional experience to apply it with no bias. Barthel Index has also been used in studies that assessed BADL as an outcome measure [36].

The intervention programme of this study is based on cognitive stimulation as a basis for the treatment of basic activities of daily living. Therefore, it is very difficult to compare it with other specific cognitive programmes whose main objective is to recover and treat the cognitive deficit.

However, the improvements in functional capacity that show an increase in the Barthel index score (*p* = 0.006) are consistent with other studies of cognitive stimulation and with recent systematic reviews [8,13,36] as well as other studies that conducted an Occupational Therapy intervention based on recovery of the ADL [34,35,37,38,39]. What makes our study different is the approach used. Their Occupational Therapy approach was based on the training of the caregivers, the modification of the environment or the use of compensatory cognitive strategies. Another aspect that makes us different is the scope of application. Most of the studies found in the literature consider Occupational Therapy intervention from a community perspective [17,18,19,35,37,38]. Therefore, none of them had institutionalised patients as participants. In contrast, their samples consisted of patients who lived at home and attended day centres or special centres for the treatment of dementia as out-patients.

Focusing on the methodology used in our cognitive stimulation programme, we can point out that the areas treated coincide with several programmes widely known in our country [40,41]. Regarding the duration and number of sessions, our intervention coincides with diverse publications that focus on the treatment of BADL [17,18,19,34,35] and Occupational Therapy [29]. The total number of sessions was 10 and they were performed twice a week for 5 weeks. Our study also coincides with other research in the field of cognitive stimulation in relation to the stage of dementia of the subjects (mild to moderate) [42,43,44,45]. It is also in accordance with other studies in relation to the cognitive scale used, the MMSE [46,47], although we used the Spanish version (Lobo’s cognitive Mini Test). Therefore, the methodology of our study is in line with other existing cognitive programmes which support and provide validity to the intervention conducted.

Authors such as Clare and Woods [27] affirmed in 2003 that individuals with mild dementia have certain capacity to learn new information or abilities if the right environmental conditions, support and patience are present. The more accessible the environment and the more training the caregivers receive, the more autonomy the patient will have [8]. This statement supports the results obtained by the Occupational Therapy ADL cognitive stimulation programme, as the participants of the intervention group improved their scores in the Barthel Index. This means that they had the capacity to learn the correct ADL performance.

In our study, the results in the experimental group were not maintained after the follow up period (Barthel Index *p* = 0.675), which could be due to the fact that the re-establishment intervention requires a longer duration in order to achieve the improvement of the cognitive function and to be applied functionally [36].

However, the basic activity that improved in the follow up measurement of our study was going upstairs and downstairs (*p* = 0.002) in the experimental group. This change could be explained by the improvement on the general independence of the participants which could have had a positive impact in their self-confidence and, in consequence, also in their motor function. Moreover, although their mobility did not improve significantly, there was an increase in the percentage of independent participants in relation to this outcome measure. At week 0 the percentage of independent participants was 57.1% and at week 5 the percentage was 60.7%. This also implied a decrease of the patients that were dependent on mobilisation.

The treatment applied, based on the cognitive stimulation, had better results than the conventional Occupational Therapy treatment (which was based on activity simplification and environmental modifications). However, although the compensatory approach in the control group did not show significant improvements in the ADLs during the five weeks of treatment, it did maintain the changes in the follow-up phase. This may be due to the fact that such an approach may require more time for the subjects to learn how to perform it, and may also serve to maintain functionality [18,34,35,38,39].

In relation to the secondary outcome measure, no significant differences in global cognitive function were observed. However, the results showed a maintenance in both groups after the intervention. We consider that this may be mainly due to the fact that the focus of the programme was on the independence to perform ADLs rather than on cognitive functions, so the sessions were not planned only for cognitive stimulation. In addition, our control group also performed cognitive stimulation, which we believe to be the reason for the lack of a statistically significant improvement. In most studies that carry out cognitive programmes, the control group either do not follow any treatment or is on the waiting list and the studies in which the control group receives any type of stimulation are very scarce [34].

Another limitation of this pilot study, in addition to the ones already mentioned, was the number of drop-outs. This was difficult to control, as the main reason for the losses was the transfer of the patients to other residential homes, a reason that was unrelated to the study itself. This could have had a negative impact on the results, not showing all the benefits that the treatments could provide to the participants after the follow-up period.

The current literature supports that non-pharmacological interventions can delay the functional deterioration of dementia, helping the patients to maintain their abilities even for one year [8,13]. Therefore, we consider that the Occupational Therapy ADL cognitive stimulation programme should be carried out for longer periods than the conventional occupational therapy treatments for the results to be maintained in time. We recommend that this consideration should be taken into account for future research.

## 5. Conclusions

Our results can be considered preliminary evidence of the feasibility of Occupational Therapy based on the training of ADL through cognitive stimulation in the independence of subjects with dementia who are institutionalised. The findings suggest a positive effect in the feeding, dressing and bladder and bowel incontinence. The changes observed in relation to feeding were statistically significant. However, the improvements were not maintained after the follow up period. Therefore, the Occupational Therapy ADL cognitive stimulation programme could be used in the clinical practice to increase the independence of these patients. However, these results need to be confirmed in a larger sample.

## Figures and Tables

**Table 1:** Baseline socio-demographic data and homogeneity of the sample.

Demographics/Clinical Data		Intervention Group			Control Group			*p*
	Category	N	%		N		%	
Gender	Male	8	28.6		5		16.7	0.277 ^a^
	Female	20	71.4		25		83.3	
Level of education (N = 57)	No studies	2	7.4		2		6.7	0.094 ^b^
	Primary School	22	81.5		28		93.3	
	Secondary School	3	11.1		0		0.0	
Year of institutionalisation	−2008	5	17.9		4		13.3	0.180 ^b^
	2009–2010	3	10.7		9		30.0	
	2011–2012	20	71.4		17		56.7	
Type of dementia	Alzheimer	4	14.3		8		2.7	0.127 ^b^
	Vascular	6	21.4		8		26.7	
	Mixed	1	3.6		2		6.7	
	Lewy body	0	0.0		2		6.7	
	Frontotemporal	0	0.0		1		3.3	
	Not specified	17	60.7		9		30.0	
Neuroleptic treatment (N = 54)	Yes	10	35.7		5		19.2	0.177 ^a^
	No	18	64.3		21		80.8	
Anxiolytic treatment (N = 54)	Yes	10	35.7		5		19.2	0.177 ^a^
	No	18	64.3		21		80.8	
Antidepressive treatment (N = 54)	Yes	6	21.4		12		46.2	0.054 ^a^
	No	22	78.6		14		53.8	
Analgesic treatment (N = 54)	Yes	0	0.0		1		3.8	0.223 ^b^
	No	28	100		25		96.2	
Other treatments of associated pathologies (N = 54)	Yes	8	28.6		5		19.2	0.422 ^a^
	No	20	71.4		21		80.8	
Occupational therapy treatment-cognitive programme	Yes	16	57.1		21		70.0	0.309 ^a^
	No	12	42.9		9		30.0	
Occupational therapy treatment-functional rehabilitation programme	Yes	11	39.3		13		43.3	0.754 ^a^
	No	17	60.7		17		56.7	
Occupational therapy treatment-ADL	Yes	28	100		30		100	*
	No	0	0.0		0		0.0	
Occupational therapy treatment-leisure	Yes	22	78.6		28		93.3	0.098 ^b^
	No	6	21.4		2		6.7	
Occupational therapy treatment- psychomotricity	Yes	5	17.9		7		23.3	0.607 ^a^
	No	23	82.1		23		76.7	
* The variable is continuous; ^a^ Pearson’s χ^2^; ^b^ Ratio of verisimilitude								
**Demographics/Clinical Data**		**Intervention Group**			**Control Group**			***p***
		**Media**		**SD**	**Media**	**SD**		
Age		84.21		7.781	81.87	6.673		0.222 ^a^
Years of dementia diagnosis		3.18		2.294	4.03	3.409		0.307 ^b^
^a^*t*-Student test, equal variances; ^b^ U of Mann-Whitney test								

Regarding the results of the Kolmogorov-Smirnov test, normality of the variable level of dependence of BADL and cognitive level can be observed (*p*-values superior to 0.05).

**Table 2:** Normality Tests.

	Global				Intervention Group				Control Group			
Outcome measure	Mean	SD	Z	*p* *	Mean	SD	Z	*p* *	Mean	SD	Z	*p* *
**Initial LCMT Test**	21.40	3.287	1.153	0.140	21.14	3.363	0.893	0.403	21.63	3.253	1.038	0.232
**Week 5 LCMT Test**	22.64	3.582	0.948	0.329	23.11	3.833	0.785	0.569	22.20	3.336	0.769	0.595
**Week12 LCMT**	21.64	3.970	0.845	0.473	21.85	4.475	0.835	0.489	21.42	3.469	0.808	0.532
**Initial Barthel Index score**	62.33	19.20	1.395	0.041	66.25	21.021	1.288	0.072	58.667	16.863	1.202	0.111
**Week 5 Barthel Index score**	65.09	20.16	1.196	0.114	72.5	20.207	1.038	0.231	58.167	17.786	1.053	0.217
**Week 12 Barthel Index score**	61.28	22.00	0.648	0.795	62.75	25.26	0.729	0.663	59.737	18.52	0.784	0.570

*p* *: *p*-value for the Kolmogorov-Smirnov test to check normality, SD: Standard deviation; Z: Z of Kolmogorov-Smirnov; LCMT: Lobo’s Cognitive Mini Test.

**Table 3:** Comparison of the Barthel Index scores after the intervention (at 5 weeks) and the severity of the dementia (GDS).

GDS at 5 Weeks	Control Group *n* = 30				Intervention Group *n* = 28			
	Mean	SD	*p*	η^2^	Mean	SD	*p*	η^2^
Absence of cognitive deficit	*	*	0.262 ^a^	0.094	90.00	0.000	0.429 ^a^	0.107
Very mild cognitive deficit	65.71	22.254			78.33	21.370		
Mild cognitive deficit	58.44	18.594			66.43	19.158		
Mild Dementia	50.00	5.000			77.14	21.575		

* No participants of the control group had absence of cognitive deficit; ^a^ ANOVA, η^2^: Effect size; GDS: Global deterioration Scale; SD: Standard deviation.

## Data Availability

The data underlying this article cannot be shared publicly to maintain the privacy of individuals that participated in the study. The data will be shared on reasonable request to the corresponding author

## Supplementary Materials

The following are available online at https://www.mdpi.com/2076-3425/11/3/319/s1, Table S1: Descriptions of the interventions conducted following the Template for Intervention. Table S2: Comparison of interventions. Table S3: Basic activities of daily living assessed with the Barthel Index that improved after the treatment.
