# Supplementary material for: Effects of a Cognitive Rehabilitation Programme on the Independence Performing Activities of Daily Living of Persons with Dementia—A Pilot Randomized Controlled Trial
Source: Brain Sci. 2021 Mar 3;11(3):319. doi: 10.3390/brainsci11030319 (PMC7998719; doi:10.3390/brainsci11030319)
Supplement: Supplementary file 1 [file brainsci-11-00319-s001.pdf]

Table S1. Descriptions of the interventions conducted following the Template for Intervention Description and Replication (TIDieR) [1]

Item nº	Item<br>**Intervention group**
**1.**	**BRIEF NAME**<br>Occupational Therapy programme based on the training of activities of daily living through cognitive stimulation.
**2.**	**WHY**<br>The programme was based on the re-establishment of the cognitive functions implied in the performance of basic activities of daily living, all cognitive stimulation activities were related to basic activities of daily living. Therefore, it was a programme focused on the rehabilitation and improvement of the basic activities of daily living and not a standard cognitive stimulation programme.
**3.**	**WHAT**<br>**Materials**: activity books, paper and pencil.
**4.**	**Procedures**: Considering the different clinical guidelines and cognitive stimulation programmes available in the literature [2,3], the cognitive functions that were trained of the intervention were the following:<br>- **First week:** Session 1: Temporal-spatial orientation, Attention /concentration and gnosia. Session 2: Temporal-spatial orientation, Attention /concentration and Praxis.<br>- **Second week**: Session 1: Temporal-spatial orientation, Attention /concentration and Executive function (planification). Session 2: Temporal-spatial orientation, Attention /concentration and Semantic memory<br>- **Third week**: Session 1: Temporal-spatial orientation, Attention /concentration and sequencing. Session 2: Temporal-spatial orientation, Attention /concentration and categorisation<br>- **Fourth week**: Session 1: Temporal-spatial orientation, Attention /concentration and language/verbal fluency. Session 2: Temporal-spatial orientation, Attention /concentration and visual discrimination<br>- **Fifth week**: Session 1: Temporal-spatial orientation, Attention /concentration and dressing praxis. Session 2: Temporal-spatial orientation, Attention /concentration and executive function<br><br>Orientation and attention were trained in all sessions as they are of prime importance for the performance of any basic activities of daily living.<br><br>The sequence of the sessions was always the same. The activities, the subject, the cognitive functions and the basic activities of daily living that were trained varied. Each session started with an activity of temporal and space orientation, continued with the performance of the specific activity of the session (that corresponded to the cognitive function to be trained in that

session) and finished with a reminiscence activity. Each activity was displayed in a sheet. This sheet showed the cognitive area and the basic activities of daily living to be trained as well as the guidelines to complete the activity.

The cognitive stimulation exercises used in the programme were:

- Orientation: activities day, date, year and representative basic activities of daily living according to the time (at what time do you eat, how many days a week do you have a shower, etc.), place where you are, community, environment and representative locations of basic activities of daily living (where is the bathroom, being able to get to the stairs or to the bedroom, etc.)
- Attention: repetition, search for elements related to basic activities of daily living that are repeated (utensils, clothes, toilet, food, bath, etc.) And circle the proposed item related to the performance of basic activities of daily living.
- Gnosia: activities of recognition of symbols, colours, objects and images that are present in the basic activities of daily living (clothes of different colours, toiletries, parts of the place where they live, eating utensils, food, etc.).
- Executive function: training in the planning of various activities of daily living, training in the sequencing of acts of daily living and daily problem solving with alleged scenarios.
- Memory: semantic memory activities related to aspects of basic activities of daily living learned throughout life.
- Praxis: activities related to the ideomotor praxis or skills: making gestures or movements of the hairstyle, dressing, climbing stairs, walking, etc. Ideological praxis or skills with manipulation of objects through a sequence of gestures.
- verbal and written language: verbal fluency and reading activities.
- categorization: activity of recognition of semantic categories of activities of daily living (types of hot or cold meals, winter and summer clothes, etc.)

	**WHO PROVIDED**
**5.**	The sessions were conducted by a qualified Occupational Therapist aid by a trained rehabilitation assistant
	**HOW**
**6.**	All the sessions were provided individually and face-to-face.
	**WHERE**
**7.**	The location were the treatment sessions were carried out was the Occupational Therapy room of the residential homes.
	**WHEN and HOW MUCH**
**8.**	The sessions lasted of 45 minutes, twice a week (Tuesday and Thursday) during 5 weeks.
	**TAILORING**
**9.**	Not applicable
	**MODIFICATIONS**
**10.**	Not applicable

	**HOW WELL**
**11.**	Intervention adherence and attendance at treatment sessions was recorded by the professional guiding the treatment.
**Item nº**	**Item**
	**Control group**
	**BRIEF NAME**
**1.**	Conventional Occupational therapy intervention for the management of activities of daily living deficits
	**WHY**
**2.**	The compensation approach was used and environment modifications and simplification of activities were applied as the intervention method.
	**WHAT**
**3.**	**Materials**: Activities of daily living materials related to the activity to be trained (clothing, toiletries, cutlery, etc.)
**4.**	**Procedures**: The activities of daily living were trained in a direct way, making the necessary adaptations to the objects used for the activities and to the activity itself, simplifying their steps or their execution.<br>The exercises were: training the use of the prescribed adaptations (thickened handle on the spoons, use of gel dispensers, clothing with velcro, etc.) or training the new way of performing the activity (learning to dry oneself using a bathrobe, drinking with a straw, dressing seated, etc.)
	**WHO PROVIDED**
**5.**	The sessions were conducted by a qualified Occupational Therapist.
	**HOW**
**6.**	All the sessions were provided individually and face-to-face.
	**WHERE**
**7.**	The location were the treatment sessions were carried out was the Occupational Therapy room of the residential homes.
	**WHEN and HOW MUCH**
**8.**	The sessions lasted of 45 minutes, twice a week (Tuesday and Thursday) during 5 weeks.
	**TAILORING**

**9.**	Not applicable
	**MODIFICATIONS**
**10.**	Not applicable
	**HOW WELL**
**11.**	Intervention adherence and attendance at treatment sessions was recorded by the professional guiding the treatment.

1. Hoffmann TC, Glasziou PP, Milne R, et al. Better reporting of interventions: Template for Intervention Description and Replication (TIDieR) checklist and guide. *BMJ* 2014; 348: g1687.
2. Tárraga L. Non-pharmacological strategy for senile brain deterioration and dementia (Estrategia no farmacológica del deterioro cerebral senil y demencia). 6th Edition. Medicine, 1994. pp.44–53 .
3. García J, Carro J. Comprehensive cognitive programme in dementia (Programa de Actuación cognitiva integral en Demencia). 1ª ed, Madrid, 2011.

Table S2. Comparison of interventions.

	INTERVENTION GROUP	CONTROL GROUP
**PROGRAME**	Occupational Therapy programme based on the training of activities of daily living through cognitive stimulation.	Conventional Occupational Therapy intervention for the management of activities of daily living deficits
**INTERVENTION METHOD**	The **re-establishment approach** of the cognitive functions implied in the performance of basic activities of daily living	The **compensation approach** was used and environment modifications and simplification of activities .
**PROCEDURES**	The cognitive functions that were trained of the intervention were the following:<br>- Temporal and space orientation<br>- Memory<br>- Attention and concentration<br>- Executive function<br>- Verbal and written language<br>- Praxis<br>- Gnosis<br>- Association and categorization.	The activities of daily living were trained in a direct way.<br><br>The exercises were:<br>- Training the use of the prescribed adaptations<br>- Training the new way of performing the activity
**PROFESSIONAL**	A qualified Occupational Therapist aid by a trained rehabilitation assistant	A qualified Occupational Therapist.

**SESSIONS**	- 45 minutes' sessions, twice a week (Tuesday and Thursday).<br>- During a period of 5 weeks.<br>- All the sessions were provided individually and face-to-face.	- 45 minutes' sessions, twice a week (Tuesday and Thursday)<br>- During a period of 5 weeks.<br>- All the sessions were provided individually and face-to-face.

Table S3. Basic activities of daily living assessed with the Barthel Index that improved after the treatment.

Outcome measure		**Intervention group**		**Control group**		*p*
		N	%	N	%	
Feeding week 5	Needs help	2	7.1	14	46.7	**0.001** [a]
	Independent	26	92.9	16	53.3	
Feeding week 12	Needs help	3	15.0	6	31.6	0.216 [b]
	Independent	17	85.0	13	68.4	
Dressing week 5	Dependent	0	0.0	4	13.3	0.005 [b]
	Needs help	16	57.1	22	73.3	
	Independent	12	42.9	4	13.3	
Dressing week 12	Dependent	6	30.0	2	10.5	0.093 [b]
	Needs help	8	40.0	14	73.7	
	Independent	6	30.0	3	15.8	
Bowels week 5	Incontinent	2	7.1	13	43.3	0.003 [a]
	Occasional episode of incontinence	6	21.4	7	23.3	
	Continent	20	71.4	10	33.3	
Bowels week 12	Incontinent	4	20.0	6	31.6	0.709 [a]
	Occasional episode of incontinence	6	30.0	5	26.3	
	Continent	10	50.0	8	42.1	
Bladder week 5	Incontinent	4	14.3	17	56.7	0.003 [a]
	Occasional episode of incontinence	9	32.1	6	20.0	

	Continent	15	53.6	7	23.3	
Bladder week 12	Incontinent	3	15.0	11	57.9	0.019 [a]
	Occasional episode of incontinence	7	35.0	4	21.1	
	Continent	10	50.0	4	21.1	
Stairs week 5	Dependent	14	50.0	16	53.3	0.157 [b]
	Needs help	9	32.1	13	43.3	
	Independent	5	17.9	1	3.3	
Stairs week 12	Dependent	14	70.0	12	63.2	**0.002** [b]
	Needs help	0	0.0	6	31.6	
	Independent	6	30.0	1	5.3	

[a] Pearson´s χ^2; [b] Likelihood ratio;

Signification level set at α=0.05/20=0.0025, using the Bonferroni correction (m=20 separate comparisons).
